# Evaluation of breast stiffness pathology based on breast compression during mammography: Proposal for novel breast stiffness scale classification

**DOI:** 10.1016/j.clinsp.2022.100100

**Published:** 2022-09-19

**Authors:** Jiří Prokop, Pavel Maršálek, Ilker Sengul, Anton Pelikán, Jana Janoutová, Petr Horyl, Jan Roman, Demet Sengul, José Maria Soares Junior

**Affiliations:** a Department of Epidemiology and Public Health, Faculty of Medicine, University of Ostrava, Czechia; b Department of Surgery, University Hospital Ostrava, Czechia; c Department of Surgical Studies, Faculty of Medicine, University of Ostrava, Czechia; d Department of Applied Mechanics, Faculty of Mechanical Engineering, VŠB-Technical University of Ostrava, Czechia; e Division of Endocrine Surgery, Faculty of Medicine, Giresun University, Turkey; f Department of General Surgery, Faculty of Medicine, Giresun University, Turkey; g Department of Health Care Sciences, Faculty of Humanities, Tomas Bata University in Zlin, Czechia; h Department of Public Health, Faculty of Medicine and Dentistry, Palacký University Olomouc, Czechia; i Department of Pathology, Faculty of Medicine, Giresun University, Turkey; j Universidade Federal de São Paulo, Faculdade de Medicina, Hospital das Clínicas, Departamento de Obstetrícia e Ginecologia, Disciplina de Ginecologia São Paulo (SP), Brasil

**Keywords:** Breast, Mammography, Stiffness, Breast pathology, Novel, Scale

## Abstract

Breast cancer is diagnosed through a patient's Breast Self-Examination (BSE), Clinical Breast Examination (CBE), or para-clinical methods. False negativity of PCM in breast cancer diagnostics leads to a persisting problem associated with breast tumors diagnosed only in advanced stages. As the tumor volume/size at which it becomes invasive is not clear, BSE and CBE play an exceedingly important role in the early diagnosis of breast cancer. The quality and effectiveness of BSE and CBE depend on several factors, among which breast stiffness is the most important one. In this study, the authors present four methods for evaluating breast stiffness pathology during mammography examination based on the outputs obtained during the breast compression process, *id est*, without exposing the patient to X-Ray radiation. Based on the subjective assessment of breast stiffness by experienced medical examiners, a novel breast stiffness classification was designed, and the best method of its objective measurement was calibrated to fit the scale. Hence, this study provides an objective tool for the identification of patients who, being unable to perform valid BSE, could benefit from an increased frequency of mammography screening. *Dum vivimus servimus.*

## Highlights

• Breast stiffness severely affects clinical- and self-examination of the breast.
• Development of subjective stiffness scale based on expert examination.
• Objective stratification based on interval of increment of strain energy density.
• Proposed scale correlates with subjective clinical examination in 92%.
• Women with stiff breasts may benefit from frequent checkup procedures.

## Introduction

Breast cancer is the most common malignant disease in women worldwide.^1^ Secondary prevention, i.e., effective screening of the disease by Para-Clinical Methods (PCM), namely Ultrasonography (US), Mammography (MMG), and Magnetic Resonance Imaging (MRI), plays a crucial role in breast cancer management; to a lesser degree, other methods, such as Scintigraphy (SG) or Elastography (ES), are utilized. The implementation of the screening program into clinical practice led to a reduction in mortality and facilitated the development of breast-sparing surgical techniques. However, the screening program comes with issues such as interval cancer; the failure to detect tumors can be revealed during Breast Self-Examination (BSE) or Clinical Breast Examination (CBE) by palpation.^2^, ^3^, ^4^ False-negative findings of screening tests can be caused by the X-Ray breast density, tumor location, or atypical X-Ray image.^5^^,^^6^ Given the possibility of such false-negative findings, BSE and CBE represent a particularly important part of a well-functioning screening program. In addition, BSE is the only examination procedure that can potentially help detect carcinoma development in younger women whose age prevents them from inclusion in the paraclinical screening program.^7^ The quality and effectiveness of BSE and CBE are, however, significantly affected by breast stiffness (mechanical stiffness of the tissue), which is given by the innate structure of the patient's tissue and influenced by the physiologic and histopathologic processes occurring in the breast tissues during life. Chronic inflammatory changes are particularly dangerous because they are associated with changes in the mammary gland architecture and coarsening of the stroma.^8^, ^9^, ^10^ Breast stiffness and X-Ray density (both characteristics are not necessarily related)^11^^,^^12^ may, therefore, increase as a consequence of such changes. At the same time, breast density and stiffness are proven risk factors for malignancy development, however, mimicry should also not be underestimated.^13^, ^14^, ^15^, ^16^, ^17^, ^18^, ^19^, ^20^ This is supported by the tissue organization field theory^21^ and the stochastic epidemiological model of tumor development.^22^, ^23^, ^24^ The measurement of tissue density has already created a corresponding sophisticated system utilized by mammography. Outcomes similar to stiffness measurements can be obtained by elastography examination.^25^^,^^26^ This type of measurement is, however, used only for certain locations inside the breast. There is a general misconception that breast stiffness closely correlates with glandular density, although this is, as mentioned above, not strictly true.^11^^,^^12^ So far, however, this field has not been extensively studied. At present, the concept of breast stiffness pathology is considered in clinical practice only marginally and even if considered, it is usually only subjectively evaluated by the examiner. Boyd et al.^27^ demonstrated that breast stiffness increases the risk of breast cancer development. These results were based on the evaluation of the statistical relationship between breast cancer and breast tissue stiffness.

The present study aims to help in the identification of patients able to perform sufficient, high-quality BSE with regard to their breast stiffness. Even though BSE was not shown to directly reduce the mortality of breast carcinoma,^28^^,^^29^ mortality is not the only endpoint in breast cancer treatment; other factors including morbidity, median survival, and quality of life, correlate well with the disease stage at diagnosis, need to be considered as well and in these outcomes, BSE was shown to play a beneficial role.^7^^,^^30^^,^^31^

The assumptions that the diagnosis of interval carcinoma is established earlier in patients with lower breast stiffness as an outcome of successful BSE and that identifying women with dense breasts during regular MMG examination would allow following up such women more frequently by CBE and paraclinical examinations, thus improving the chance for early detection of breast carcinoma, are logical; nevertheless, such hypotheses have not been sufficiently investigated yet. One of the principal reasons for this is the fact that there is currently no objective method for breast stiffness measurement. The method proposed in this paper would allow such research and confirmation (or disproval) of this hypothesis on a larger population scale. This study describes four methods of evaluating data obtained during compression of the breast during a standard MMG examination and compares the results with CBE.

## Materials and methods

### Characteristics of subjects and design of stiffness scale

In 2016 and 2017, one hundred Caucasian women examined in a mammography unit at the Department of Surgery, University Hospital Ostrava, Czech were asked to participate in the study. On random days at the outpatient mammography clinic (if the clinic workload permitted), all patients attending the clinic who were eligible for inclusion were offered participation in the study. A total of 100 females were approached based on the inclusion criteria (free of tumor at present as well as in the personal history, inflammation, and any other form of breast disease, without prior surgical intervention or evolutionary breast anomaly). All patients have consented to be included in the study; none have declined the inclusion. Ten women had to be removed from the study due to the recording device malfunction. Provided the women were hormonally active, measurements had to be carried out between the 3^rd^ and 10^th^ day after menstruation. The exclusion criteria included the use of hormonal saturation during menopause and a period of fewer than two years since the last time they breastfed. All the subjects included in the present study signed the informed consent. The study was approved by the Ethics Committee of the University Hospital of Ostrava, Ostrava, Czechia. All the cases had been treated according to the principles of the Declaration of Helsinki.

The CBE was performed on each case, independently by two experienced examiners working at the University Hospital Ostrava on the same day. The examiners have over 30 years of experience in Breast Surgery both in the outpatient and surgical settings. The outcomes of breast stiffness measurement as measured by MMG were blinded to both examiners until the end of the study.

Subsequently, on the same visit, MMG examinations of the cases were carried out, with special attention paid to the initial compression process that can be easily automated and, thus, used for objective evaluation of breast stiffness (see hereinafter for more details). During this process, the breast is compressed by the upper paddle. Herein, the force required for the compression was measured and used to calculate breast stiffness (see below), which was eventually compared to that determined by the physicians. The resulting MMG measurements were blinded to both examiners until the final data evaluation.

The mean patients' age range on the examinations was 54.8±11.7 years (min. 32 years, Patient 65; max. 83 years, Patient 42). The patients with all breast sizes were included in the study; the mean body mass index range was 27.4±4.45 (min. 19.7, Patient 12; max 38.1, Patient 1).^32^ The statistical analysis, as well as the creation of a model for evaluation of breast stiffness, were performed in MATLAB (MathWorks, Natick, MA, USA).

### Design of the stiffness scale

To classify patients in terms of their breast stiffness pathology, a novel stiffness scale was designed. Proposal for novelty via this new linear scale indicates the suitability of CBE or BSE methods for examination based on the palpation perception and is divided into five stiffness classes where lower classes (Class I, II, and III) indicate that the breast is sufficiently examinable by palpation. Class I indicates the most transparent examination (easy to examine), and Class II and III represent well-examinable and sufficiently examinable breasts, respectively. Class IV means that the breast examination by palpation is difficult and Class V indicates non-examinable breast. The patients in the last two classes are unable to perform a valid BSE and are, therefore, eligible for more frequent paraclinical observation and the use of other examination methods, such as US or MRI. The classification according to the novel stiffness scale is exhibited in Table 1.

**Table 1:** Distribution of patients into the stiffness classes.

Class	Description
I	Easy to examine
II	Well-examinable
III	Sufficiently examinable
IV	Difficult to examine
V	Non-examinable

Patients with all breast types present in the general population were included in this study, which facilitated the testing of the robustness of the measurement method under development. All the measurements and examinations were performed on the right breast. The distribution of patients in individual classes is shown in Table 1.

### Methods of the compression of the breast during MMG examination

**Figure 1:**
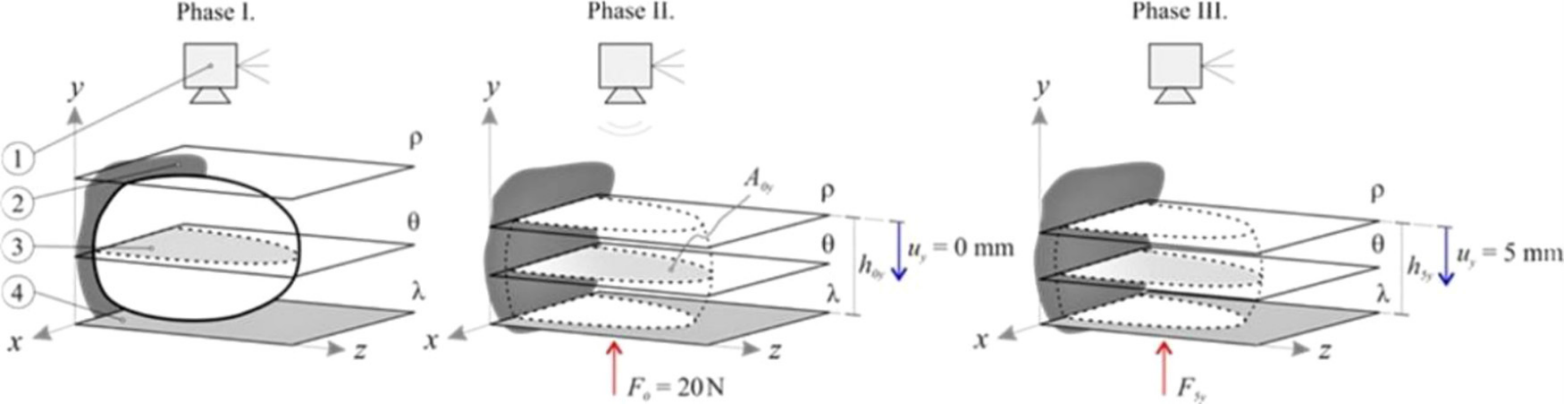
Methodology of compression of the breast in the vertical direction during the MMG examination (1: camera, 2: top MMG paddle, 3: breast cross-section area, 4: bottom MMG paddle, ρ: moving plane representing the top MMG paddle, θ: parallel plane indicating the breast cross-section area, λ: fixed plane corresponding to the bottom MMG paddle).

For measurement purposes, a fully digital mammography Mammomat Inspiration (MI; Siemens, Munich, Germany) used at the University Hospital Ostrava had been utilized. During the MMG examination, the breast was placed between the MMG paddles to enable the compression of the breast. Figure 1 presents the methodology of MMG examination in the vertical direction, where λ represents the bottom MMG paddle (Item 4 in Fig. 1) and ρ is a moving plane representing the top MMG paddle (Item 2 in Fig. 1). The movement of the ρ plane indicates breast compression.

The MMG paddles were selected to simulate the palpation examination of breast stiffness. Based on the authors’ experiences, the breast manipulation during the MMG (where the breast is placed between the MMG paddles) very well imitates the expert palpation examination (CBE method). For breast stiffness evaluation during MMG, only the first two out of three standard MMG compression phases described below are required.

The first phase (Phase I) describes the initial process of breast compression and stabilization of contact areas between the breast and the MMG paddles (Fig. 1). To enable the examination of both stiff and soft breasts, breast preload was set to *F_0_* = 20 N for all the measurements and directions. Upon reaching the breast preload, the distance between the paddles at the moment of achieving breast preload had been measured and considered baseline (*h_0i_*, i.e., *h_0y_* during vertical measurement, *h_0x_* during horizontal measurement, respectively).

**Figure 2:**
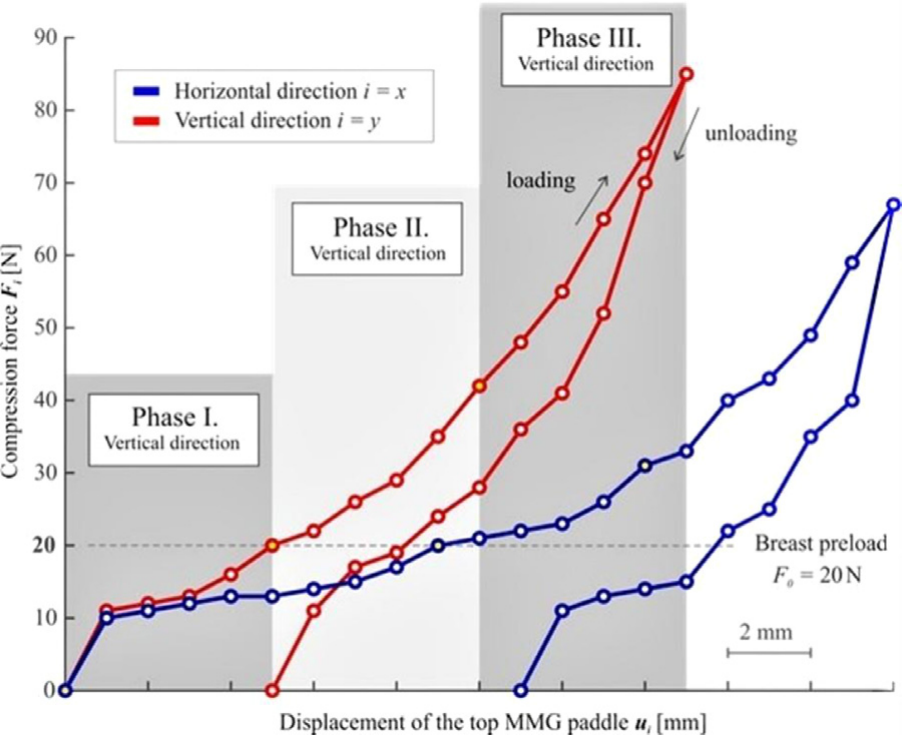
Typical non-linear characteristics of the breast compression during MMG examination – the relationship between the compression force F_i_ and displacement of the top MMG paddle u_i_ for the horizontal and vertical direction.

The main phase follows (Phase II), at the beginning of which a photograph is taken (which is necessary for the subsequent analysis of the breast cross-section area *A_0i_* that is needed for some of the evaluation methods). Afterward, the compression measurement, *per se*, initiates. The compression is elicited by further downward movement (displacement) of the top MMG paddle (Fig. 1). To facilitate the examination of small as well as large breasts, it was necessary to design an ideal, universal, compression interval. The top paddle displacement value of *u_max_* = 5 mm has been established based on practical measurement experience (a value at which no significant change in breast shape was observed). The use of this interval facilitates the examination of both small and large breasts without causing significant discomfort to the patients. The recorded discrete relationship between the compression forces F_i_ and the displacement of the top MMG paddle u_i_ for both orthogonal planes are presented in Figure 2, showing the non-linear dependency of the compression paddle response (i.e., the dependence of the force on the displacement) with significant hysteresis. No breast irradiation is used in this method. Controlled compression was defined using the step size of *u* = 1 mm with the tolerance of Δ*u* = 0.1 mm and the force after each step was measured with an accuracy of Δ*F* = 1 N. At the end of Phase II, the maximal compression force *F_5i_* was measured, corresponding to the final distance between the MMG paddles *u_max_* = 5 mm. The last phase (Phase III) of the standard MMG examination, during which the breast is further compressed to achieve compression suitable for breast irradiation and irradiation is used, is irrelevant for the purposes of the breast stiffness measurement and so is the breast unloading after the X-Ray (as shown in Figure 2, the relationship between the compression force and the paddle displacement during unloading differs from that in the compression phase).

The photograph obtained at the beginning of Phase II was post-processed using Fiji software^15^ in order to analyze the initial breast cross-section area *A_0i_*. The post-processing of the photograph was not automated. Each record was manually calibrated, and the initial breast cross-section was manually delineated.

The initial breast volume *V_0i_* for each direction was calculated using the equation: $V_{0i}=A_{0i}\;h_{0i}$; where *A_0i_* represents the breast cross-section area and *h_0i_* is the initial distance between the MMG paddles.

### Methods of the breast evaluation during MMG examination

Evaluation of breast stiffness using the measured relationship between the compression force *F_i_* and the top MMG paddle displacement *u_i_* was analyzed by four methods presented below, namely: (i) Boyd's radial stiffness, (ii) linearized stiffness, (iii) calculation of the elastic modulus and (iv) calculation of the increment of strain energy density.

### Evaluation of Boyd's radial stiffness

**Figure 3:**
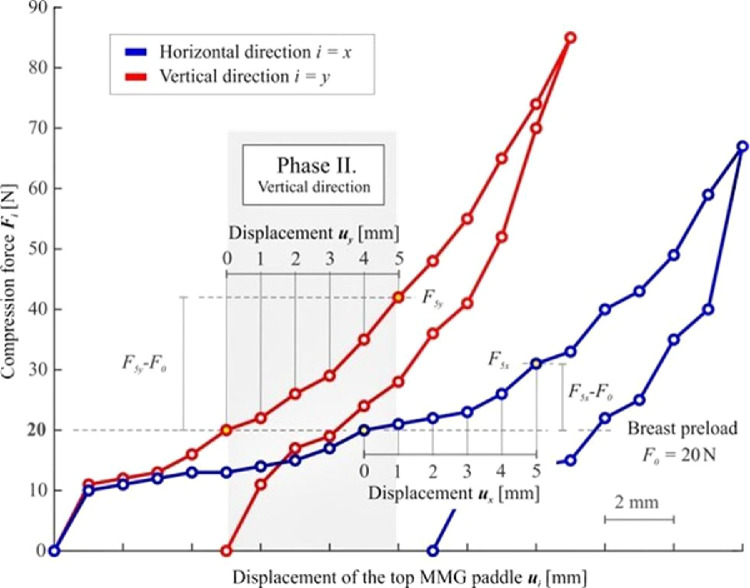
Evaluation of Boyd's radial stiffness *k_Bi_* in the interval of the displacement of *u_i_* = 0-5 mm.

The first method for stiffness evaluation is Boyd's radial stiffness *k_Bi_*, which has been adopted from a previously published paper by Boyd et al.^16^ Boyd's evaluation^16^ is based on the hemispheric idealization of the breast shape with a radius *r*. The detected radiuses before compression *r_0i_* (corresponding to the breast preload *F_0_*) and *r_5i_* after compression (corresponding to *F_5i_* detected at the final distance between MMG paddles) had been used to describe the changes in the breast shape (Fig. 3). Boyd's radial stiffness for each direction is described by the equation $k_{Bi}=\frac{F_{5i}-F_{0}}{r_{0i}-r_{5i}}$; with the required initial radius *r_0i_* obtained from the equation for an idealized semicircular area $A_{0i}=\frac{1}{2}\pi r_{0i}^{2}$; where *A_0i_* represents the analyzed initial breast cross-section area. The final radius *r_5i_* is derived using the final breast volume after compression idealized as a hemispherical shape $V_{5i}=\frac{1}{2}\left(\frac{4}{3}\pi r_{5i}^{3}\right)$; where the final breast volume *V_5i_* (after compression) is calculated using the equation $V_{5i}=A_{0i}\left(h_{0i}-u_{max}\right)$.

### Evaluation of linearized stiffness

**Figure 4:**
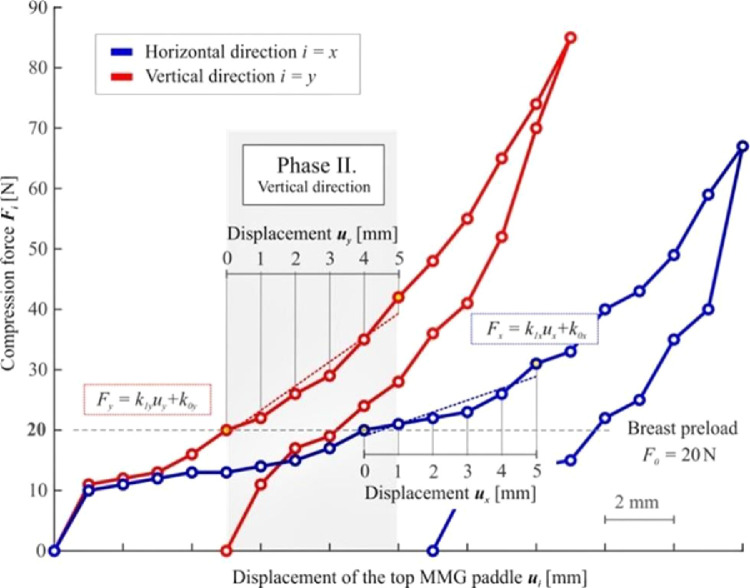
Evaluation of the linearized stiffness *k_1i_* in the interval of the displacement of *u_i_* = 0-5 mm.

The second method for analyzing breast stiffness lies in the linear approximation of the measured response using the least-squares method in the interval of displacement of *u_i_* = 0‒5 mm, see Figure 4. The linearized stiffness approximates the dependence of the force applied by the top MMG paddle on its displacement, disregarding the breast geometry.

The master equation for the force *F_i_* is given by $F_{i}=k_{1i}u_{i}+k_{0i}$; where important coefficients *k_1i_* can be calculated as $k_{1i}=\frac{6\left(\sum_{p=0}^{5}F_{ip}u_{ip}\right)-\left(\sum_{p=0}^{5}F_{ip}\right)\cdot \left(\sum_{p=0}^{5}u_{ip}\right)}{6\left(\sum_{p=0}^{5}{u_{ip}}^{2}\right)-{\left(\sum_{p=0}^{5}u_{ip}\right)}^{2}}$; in the interval of the displacement of *u_i_* = 0‒5 mm. This calculation yields two coefficients *k_1i_* (one for each direction) representing the linearized breast stiffness.

### Evaluation of elastic modulus

The third method the authors used for analyzing breast stiffness was the determination of the elastic modulus (a global value for the whole breast). For simplification, homogenous and isotropic behavior of the breast was considered and the authors were aware that these assumptions are not based on real breast behavior because the breast consists of numerous tissues with different qualities. However, the authors performed the analysis only in a small, well-defined interval of displacement of *u_i_* = 0-5 mm, which allowed us to perform such an elasticity evaluation.

Assuming the constant breast cross-section area A_0i_ and small deformation, linearized stiffness (Equation 7) can be expressed using the equation $k_{1i}=\frac{E_{i}A_{0i}}{h_{0i}}$; where *E_i_* represents the elastic modulus and *h_0i_* is the initial distance between the MMG paddles obtained from Table 1.

### Evaluation of the increment of strain energy density

**Figure 5:**
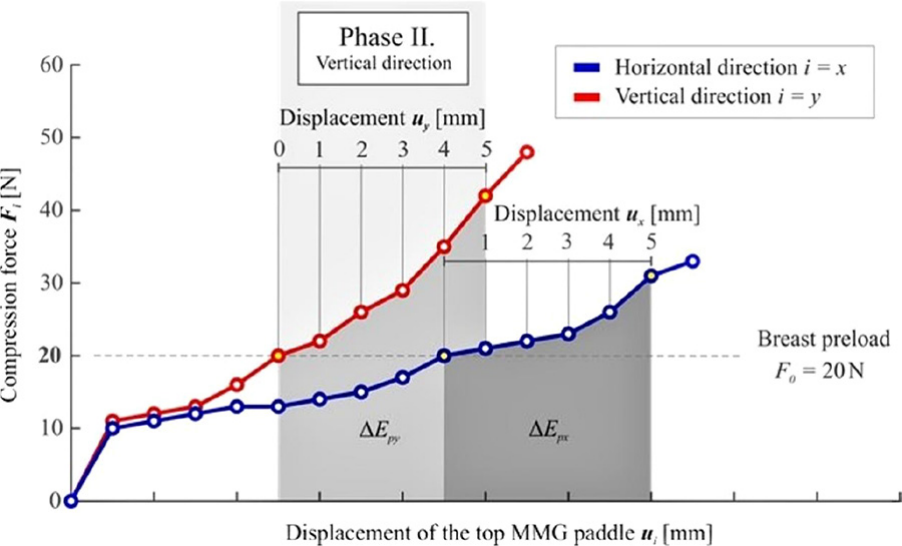
Increments of the strain energy Δ*E_pi_* in the interval of the displacement of *u_i_* = 0-5 mm.

The energetic approach evaluates the increment of strain energy density used to compress the breast volume defined by the equation $\Delta U_{i}=\frac{\Delta E_{pi}}{\Delta V_{i}}$; where Δ*E_pi_* is the increment of strain energy and Δ*V_i_* represents the change of the breast volume during compression. To establish Δ*E_pi_*, a numeric integration of the curve describing the non-linear response of the compression force *F_i_* to the displacement of the top MMG paddle *u_i_* is performed, see Figure 5.

The increment of strain energy is again evaluated in the interval of displacement *u_i_* = 0‒5 mm using the equation $\Delta E_{pi}=\int_{0}^{u_{max}}F_{i}du_{i}=\sum_{n=1}^{5}\frac{1}{2}\left(F_{i_{n-1}}+F_{i_{n}}\right)u_{i_{n}}$.

For determination of the change of breast volume Δ*V_i_*, the following equation is used $\Delta V_{i}=V_{0i}-V_{5i}=A_{0i}h_{0i}-A_{0i}\left(h_{0i}-u_{max}\right)=A_{0i}u_{max}$; where the final breast volume *V_5i_* (after compression) is subtracted from the initial volume *V_0i_* defined by Equation 1. It was presumed and confirmed by measurement that during the compression by *u_max_* = 5 mm, no significant change of the initial breast cross-section area *A_0i_* occurs.

## Results

This section presents the results of the individual methods divided into three chapters. In Chapter 3.1, the results obtained by CBE performed independently by two experienced examiners will be presented while Chapter 3.2 describes the results measured by individual instrument-based approaches and compares their effectiveness. Of note, Chapter 3.3 reveals the relevant outcomes of the best of these approaches are compared with those obtained by CBE.

### Breast stiffness is classified based on CBE according to the novel stiffness scale

Results of CBE testing for individual patients are shown in Table 2. Patients highlighted in bold (12, 18, 27, 56, 59, 62, 72, 77, 78, 79, 80, 82, and 85) were classified as difficult to examine or non-examinable, i.e., as belonging to Classes IV and V, respectively. Seventy-seven cases in the present study design were classified as Classes I‒III (i.e., as patients in whom self-examination should pose no problem), 12 as Class IV (BSE difficult), and in one case, BSE was impossible (Class V).

**Table 2:** Breast stiffness is classified based on CBE according to the novel stiffness scale, initial breast cross-section area *A_0i,_* initial distance between mammographic paddles *h_0i,_* and initial breast volume *V_0i_* corresponding to the breast preload *F_0_* (horizontal direction *I* = *x* and vertical direction *I* = *y)*. Patients with the highest breast stiffness (Classes IV and V) are highlighted in bold italics.

Patient number [-]	Class, based on CBE [-]	*A_0y_* [mm^2^]	*A_0x_* [mm^2^]	*h_0y_* [mm]	*h_0x_* [mm]	*V_0y_* [mm^3^]	*V_0x_* [mm^3^]
1	1	1.687·10^4^	1.747·10^4^	82	76	1.383·10^6^	1.328·10^6^
2	2	1.694·10^4^	1.699·10^4^	100	79	1.694·10^6^	1.342·10^6^
3	3	1.211·10^4^	1.280·10^4^	61	56	8.609·10^5^	7.170·10^5^
4	1	1.395·10^4^	1.073·10^4^	107	63	1.493·10^6^	7.390·10^5^
5	2	1.973·10^4^	1.549·10^4^	84	70	1.657·10^6^	1.085·10^6^
6	3	9.436·10^3^	9.015·10^3^	50	56	4.718·10^5^	5.048·10^5^
7	2	1.549·10^4^	1.783·10^4^	58	52	8.982·10^5^	9.272·10^5^
8	2	1.426·10^4^	9.163·10^3^	105	74	1.497·10^6^	6.781·10^5^
9	3	7.792·10^3^	8.523·10^3^	52	55	4.052·10^5^	4.688·10^5^
10	2	1.446·10^4^	1.111·10^4^	71	57	1.027·10^6^	6.330·10^5^
11	3	5.512·10^3^	7.234·10^3^	73	79	4.024·10^5^	5.715·10^5^
**12**	**4**	**6.202·10^3^**	**6.843·10^3^**	**56**	**43**	**3.473·10^5^**	**2.942·10^5^**
13	2	1.959·10^4^	1.508·10^4^	79	69	1.548·10^6^	1.041·10^6^
14	1	1.759·10^4^	1.177·10^4^	111	80	1.952·10^6^	1.101·10^6^
15	3	8.379·10^3^	1.099·10^4^	52	57	4.357·10^5^	6.265·10^5^
16	2	1.033·10^4^	1.129·10^4^	74	77	7.644·10^5^	8.692·10^5^
17	3	8.260·10^3^	9.269·10^3^	61	60	5.039·10^5^	5.561·10^5^
**18**	**4**	**5.052·10^3^**	**6.448·10^3^**	**66**	**60**	**3.334·10^5^**	**3.869·10^5^**
19	1	2.715·10^4^	2.038·10^4^	85	66	2.307·10^6^	1.345·10^6^
20	1	2.003·10^4^	2.311·10^4^	76	75	1.522·10^6^	1.733·10^6^
21	3	1.686·10^4^	1.565·10^4^	86	68	1.450·10^6^	1.064·10^6^
22	3	1.186·10^4^	6.278·10^3^	59	54	6.996·10^5^	3.390·10^5^
23	2	1.806·10^4^	1.406·10^4^	49	43	8.848·10^5^	6.045·10^5^
24	1	2.544·10^4^	2.485·10^4^	86	80	2.188·10^6^	1.988·10^6^
25	3	7.952·10^3^	9.624·10^3^	60	58	4.771·10^5^	5.582·10^5^
26	2	1.154·10^4^	1.210·10^4^	63	63	7.270·10^5^	7.620·10^5^
**27**	**4**	**8.064·10^3^**	**6.881·10^3^**	**79**	**67**	**6.371·10^5^**	**4.610·10^5^**
28	3	1.061·10^4^	1.013·10^4^	78	68	8.276·10^5^	6.888·10^5^
29	2	1.087·10^4^	9.701·10^3^	80	73	8.693·10^5^	7.082·10^5^
30	2	1.314·10^4^	1.211·10^4^	71	63	9.326·10^5^	7.627·10^5^
31	2	6.637·10^3^	8.691·10^3^	60	54	3.982·10^5^	4.693·10^5^
32	2	1.086·10^4^	1.509·10^4^	72	66	7.819·10^5^	9.960·10^5^
33	2	1.090·10^4^	1.386·10^4^	69	67	7.523·10^5^	9.283·10^5^
34	2	1.136·10^4^	1.467·10^4^	54	63	6.137·10^5^	9.243·10^5^
35	3	1.263·10^4^	1.276·10^4^	90	67	1.137·10^6^	8.548·10^5^
36	2	1.949·10^4^	1.478·10^4^	78	69	1.520·10^6^	1.020·10^6^
37	3	9.687·10^3^	1.563·10^4^	76	69	7.362·10^5^	1.079·10^6^
38	3	7.081·10^3^	9.351·10^3^	54	53	3.824·10^5^	4.956·10^5^
39	2	2.015·10^4^	1.819·10^4^	63	53	1.270·10^6^	9.642·10^5^
40	3	8.856·10^3^	1.073·10^4^	52	51	4.605·10^5^	5.472·10^5^
41	2	1.997·10^4^	1.954·10^4^	95	69	1.897·10^6^	1.348·10^6^
42	2	1.777·10^4^	1.676·10^4^	62	63	1.102·10^6^	1.056·10^6^
43	3	8.375·10^3^	1.045·10^4^	73	62	6.114·10^5^	6.481·10^5^
44	3	1.346·10^4^	1.214·10^4^	69	54	9.285·10^5^	6.553·10^5^
45	3	8.366·10^3^	9.302·10^3^	56	59	4.685·10^5^	5.488·10^5^
46	2	1.918·10^4^	1.727·10^4^	58	50	1.113·10^6^	8.635·10^5^
47	3	8.326·10^3^	1.021·10^4^	63	72	5.245·10^5^	7.354·10^5^
48	2	9.092·10^3^	1.188·10^4^	83	64	7.546·10^5^	7.603·10^5^
49	3	9.399·10^3^	1.457·10^4^	69	70	6.485·10^5^	1.020·10^6^
50	2	1.050·10^4^	1.082·10^4^	56	58	5.877·10^5^	6.274·10^5^
51	3	1.601·10^4^	1.544·10^4^	71	69	1.136·10^6^	1.065·10^6^
52	2	1.366·10^4^	1.366·10^4^	95	85	1.298·10^6^	1.161·10^6^
53	2	1.474·10^4^	1.340·10^4^	62	56	9.137·10^5^	7.502·10^5^
54	2	1.147·10^4^	1.049·10^4^	67	71	7.685·10^5^	7.451·10^5^
55	3	1.001·10^4^	1.085·10^4^	57	59	5.708·10^5^	6.402·10^5^
**56**	**4**	**5.317·10^3^**	**4.362·10^3^**	**40**	**57**	**2.127·10^5^**	**2.486·10^5^**
57	3	1.113·10^4^	1.259·10^4^	54	50	6.010·10^5^	6.294·10^5^
58	3	1.201·10^4^	1.046·10^4^	70	73	8.406·10^5^	7.634·10^5^
**59**	**4**	**8.148·10^3^**	**5.642·10^3^**	**42**	**55**	**3.422·10^5^**	**3.103·10^5^**
60	3	8.510·10^3^	9.000·10^3^	50	57	4.255·10^5^	5.130·10^5^
61	2	2.503·10^4^	2.650·10^4^	69	57	1.727·10^6^	1.510·10^6^
**62**	**4**	**4.112·10^3^**	**8.512·10^3^**	**70**	**59**	**2.878·10^5^**	**5.022·10^5^**
63	3	9.197·10^3^	1.031·10^4^	51	67	4.690·10^5^	6.908·10^5^
64	2	1.272·10^4^	1.126·10^4^	59	73	7.504·10^5^	8.219·10^5^
65	2	1.123·10^4^	1.251·10^4^	52	52	5.840·10^5^	6.506·10^5^
66	3	1.008·10^4^	9.926·10^3^	63	67	6.351·10^5^	6.650·10^5^
67	2	1.416·10^4^	1.500·10^4^	88	69	1.246·10^6^	1.035·10^6^
68	1	1.821·10^4^	2.579·10^4^	66	52	1.202·10^6^	1.341·10^6^
69	3	1.257·10^4^	1.275·10^4^	69	62	8.675·10^5^	7.906·10^5^
70	3	6.525·10^3^	7.162·10^3^	37	35	2.414·10^5^	2.507·10^5^
71	2	1.858·10^4^	1.986·10^4^	69	61	1.282·10^6^	1.211·10^6^
**72**	**4**	**5.839·10^3^**	**6.007·10^3^**	**72**	**90**	**4.204·10^5^**	**5.406·10^5^**
73	3	1.019·10^4^	9.668·10^3^	59	53	6.013·10^5^	5.124·10^5^
74	2	1.437·10^4^	2.281·10^4^	114	79	1.638·10^6^	1.802·10^6^
75	1	9.401·10^3^	1.052·10^4^	48	58	4.512·10^5^	6.100·10^5^
76	2	2.277·10^4^	2.448·10^4^	76	65	1.731·10^6^	1.591·10^6^
**77**	**4**	**4.323·10^3^**	**8.387·10^3^**	**77**	**59**	**3.329·10^5^**	**4.948·10^5^**
**78**	**4**	**5.764·10^3^**	**6.629·10^3^**	**75**	**72**	**4.323·10^5^**	**4.773·10^5^**
**79**	**4**	**5.684·10^3^**	**6.116·10^3^**	**71**	**69**	**4.036·10^5^**	**4.220·10^5^**
**80**	**4**	**5.681·10^3^**	**5.024·10^3^**	**47**	**53**	**2.670·10^5^**	**2.663·10^5^**
81	3	8.402·10^3^	1.239·10^4^	61	55	7.565·10^5^	7.912·10^5^
**82**	**4**	**4.559·10^3^**	**6.877·10^3^**	**58**	**61**	**2.644·10^5^**	**4.195·10^5^**
83	2	1.097·10^4^	1.443·10^4^	59	45	6.471·10^5^	6.492·10^5^
84	2	1.240·10^4^	1.321·10^4^	65	63	8.060·10^5^	8.321·10^5^
**85**	**5**	**6.166·10^3^**	**5.419·10^3^**	**35**	**32**	**2.158·10^5^**	**1.734·10^5^**
86	2	1.946·10^4^	1.823·10^4^	62	48	1.206·10^6^	8.750·10^5^
87	3	1.145·10^4^	7.949·10^3^	45	47	5.152·10^5^	3.736·10^5^
88	3	1.047·10^4^	1.518·10^4^	56	57	5.861·10^5^	8.653·10^5^
89	2	1.389·10^4^	1.480·10^4^	75	58	1.041·10^6^	8.581·10^5^
90	2	1.095·10^4^	1.205·10^4^	49	49	5.366·10^5^	5.906·10^5^

### Results of the evaluation of the breast during the MMG examination

This section will be divided into five sub-chapters presenting the breast size of individual patients and the results of the four methods of stiffness determination.

### Evaluation of the breast size

The analyzed patients' initial breast cross-section area *A_0i_* at the breast preload *F_0_* for both directions are depicted in Table 2. The mean patients' initial breast cross-section area was 1.232·10^4^ ± 0.480·10^4^ mm^2^ (min. 0.484·10^4^ mm^2^, Patient 56; max. 2.576·10^4^ mm^2^, Patient 61; note that the mean from both directions was considered). The minimum difference between the vertical and horizontal direction in the same patient was 0.001·10^4^ mm^2^ (Patient 52), and the maximum difference was 0.844·10^4^ mm^2^ (Patient 74).

The initial distance between the MMG paddles *h_0i_* corresponding to the initial breast thickness was captured in all patients (Table 2). The mean distance (i.e., mean from the *x* and *y* directions) between the MMG paddles was 64.7±12.3 mm (min. 33.5 mm, Patient 85; max. 96.5 mm, Patient 74). The minimum difference between the vertical and horizontal direction in the same patient was 0 mm (Patients 26, 65, and 90), and the maximum difference was 44.0 mm (Patient 4).

The mean patients' initial breast volume calculated using Equation 1 was 8.222·10^5^ ± 4.041·10^5^ mm^3^ (min. 1.946·10^5^ mm^3^, Patient 85; max. 20.88·10^5^ mm^3^; Patient 24; note that the mean from both directions was considered), see Table 2. The minimum difference between the vertical and horizontal direction in the same patient was 0.007·10^5^ mm^3^ (Patient 80), and the maximum difference was 9.623·10^5^ mm^3^ (Patient 19).

### Evaluation of Boyd's radial stiffness

Evaluation of Boyd's radial stiffness *k_Bi_* in the interval of the displacement of *u_i_* = 0‒5 mm for both directions are shown in Table 3. The mean Boyd's radial stiffness in the present patient group was 1.031±1.500 N·mm^−1^ (min. -9.929 N·mm^−1^, Patient 72; max. 5.035 N·mm^−1^, Patient 78; note that the mean from both directions was considered). The minimum difference between the vertical and horizontal direction in the same patient was 0.041 N·mm^−1^ (Patient 63), and the maximum difference was 27.74 N·mm^−1^ (Patient 72). The obtained results exhibit negative stiffness values for Patients 72 and 77. This was caused by the negative difference between the initial and final calculated radiuses (*r_0i_* - *r_5i_* < 0), which was caused by the inadequate geometric assumption, indicating that the shape of breasts cannot be considered hemispherical in all cases. In Patient 29, the forces *F_5y_* and *F_0y_* in the vertical direction are the same (although the forces measured between these limiting states are different) due to the fluctuation in the breast resistance. This, in effect, would cause the Boyd radial stiffness in the vertical direction to be *k_by_* = 0 N·mm^−1^. In view of these results, this approach seems imperfect and not universally applicable to all patients.

**Table 3:** Breast stiffness is classified based on CBE according to the newly designed stiffness scale, evaluation of Boyd's radial stiffness *k_Bi_*, linearized stiffness *k_1i,_* and the elastic modulus *E_i_*. All the calculated values are calculated in the interval of the displacement of *u_i_* = 0‒5 mm (horizontal direction *I* = *x* and vertical direction *i* = *y)*.

Patient number [-]	Class, based on CBE [-]	*k_By_* [N·mm^−1^]	*k_Bx_* [N·mm^−1^]	*k_1y_* [N·mm^−1^]	*k_1x_* [N·mm^−1^]	*E_y_* [N·mm^−2^]	*E_x_* [N·mm^−2^]
1	1	0.558	0.491	2.400	1.743	1.044·10^−2^	8.473·10^−3^
2	2	1.527	0.326	6.029	0.971	2.803·10^−2^	5.734·10^−3^
3	3	1.557	0.755	6.514	3.171	2.849·10^−2^	1.371·10^−2^
4	1	1.358	0.790	4.657	0.714	2.501·10^−2^	5.478·10^−3^
5	2	0.858	1.165	4.000	5.314	1.807·10^−2^	2.262·10^−2^
6	3	1.750	1.066	5.171	4.286	3.212·10^−2^	2.271·10^−2^
7	2	1.309	0.689	7.857	3.429	2.291·10^−2^	1.284·10^−2^
8	2	1.506	1.916	3.286	2.743	2.654·10^−2^	2.020·10^−2^
9	3	1.617	0.896	5.200	2.971	3.356·10^−2^	1.983·10^−2^
10	2	1.579	0.632	5.771	2.400	2.962·10^−2^	1.179·10^−2^
11	3	3.636	3.468	2.857	1.971	3.120·10^−2^	2.611·10^−2^
12	4	2.172	1.252	6.800	2.514	4.273·10^−2^	2.270·10^−2^
13	2	0.723	0.560	3.086	2.600	1.412·10^−2^	1.048·10^−2^
14	1	0.889	0.312	2.600	0.600	1.511·10^−2^	3.787·10^−3^
15	3	0.585	2.223	2.657	7.886	1.378·10^−2^	4.894·10^−2^
16	2	2.620	0.887	6.600	1.829	4.502·10^−2^	1.310·10^−2^
17	3	2.156	1.160	5.629	2.800	3.643·10^−2^	2.068·10^−2^
18	4	3.544	1.276	5.629	1.229	5.238·10^−2^	1.605·10^−2^
19	1	1.005	0.033	5.886	0.114	1.906·10^−2^	3.578·10^−4^
20	1	0.339	0.280	1.829	1.543	5.935·10^−3^	5.855·10^−3^
21	3	2.134	0.474	8.429	1.429	3.662·10^−2^	7.287·10^−3^
22	3	2.677	0.666	5.429	2.514	4.669·10^−2^	1.251·10^−2^
23	2	1.283	0.373	7.743	2.371	2.368·10^−2^	6.435·10^−3^
**24**	**1**	**0.642**	**0.683**	**4.029**	**3.486**	**1.297·10^−2^**	**1.178·10^−2^**
25	3	1.580	0.930	5.200	2.343	3.134·10^−2^	1.768·10^−2^
26	2	1.311	0.577	4.857	2.057	2.530·10^−2^	1.123·10^−2^
27	4	4.219	2.223	6.057	2.629	5.898·10^−2^	2.575·10^−2^
28	3	0.845	0.769	2.229	1.657	1.496·10^−2^	1.218·10^−2^
**29**	**2**	**1.422**	**0.000**	**3.286**	**0.229**	**2.472·10^−2^**	**1.683·10^−3^**
30	2	1.637	0.355	5.686	0.943	2.959·10^−2^	5.097·10^−3^
31	2	2.632	1.203	8.257	2.400	5.130·10^−2^	2.170·10^−2^
32	2	0.773	1.245	3.457	3.200	1.512·10^−2^	2.122·10^−2^
33	2	1.121	0.929	4.257	2.371	2.059·10^−2^	1.501·10^−2^
34	2	0.886	1.349	3.771	5.229	1.620·10^−2^	2.485·10^−2^
35	3	2.635	1.349	9.571	2.314	5.027·10^−2^	1.649·10^−2^
36	2	0.789	0.468	3.371	2.314	1.574·10^−2^	9.262·10^−3^
37	3	0.648	1.153	2.971	2.257	1.312·10^−2^	1.771·10^−2^
38	3	1.040	0.737	3.543	1.971	2.008·10^−2^	1.503·10^−2^
39	2	0.547	0.711	3.543	4.400	1.032·10^−2^	1.376·10^−2^
40	3	1.341	1.255	6.114	3.829	2.906·10^−2^	2.248·10^−2^
41	2	1.208	0.847	6.114	2.743	2.159·10^−2^	1.305·10^−2^
42	2	1.121	0.789	6.457	3.971	2.427·10^−2^	1.385·10^−2^
43	3	1.708	1.093	5.229	1.771	3.101·10^−2^	1.544·10^−2^
44	3	1.263	0.440	5.429	2.029	2.416·10^−2^	1.040·10^−2^
45	3	1.956	0.849	5.829	2.371	3.697·10^−2^	1.587·10^−2^
46	2	1.515	0.595	8.829	3.371	2.556·10^−2^	1.019·10^−2^
47	3	1.275	1.132	2.829	2.629	1.994·10^−2^	1.989·10^−2^
48	2	1.255	0.470	4.800	0.229	2.586·10^−2^	2.087·10^−3^
49	3	1.279	1.055	4.771	2.286	2.293·10^−2^	1.678·10^−2^
50	2	1.108	1.039	4.171	3.457	2.237·10^−2^	1.845·10^−2^
51	3	0.940	0.469	3.914	1.886	1.750·10^−2^	8.365·10^−3^
52	2	1.020	0.315	2.800	0.486	1.743·10^−2^	3.377·10^−3^
53	2	1.021	0.389	4.943	1.771	2.066·10^−2^	7.453·10^−3^
54	2	1.589	0.701	4.429	2.114	2.996·10^−2^	1.235·10^−2^
55	3	0.847	0.943	3.000	3.571	1.631·10^−2^	2.033·10^−2^
56	4	1.789	1.180	1.743	3.229	2.277·10^−2^	2.429·10^−2^
57	3	0.926	1.526	4.514	6.200	1.793·10^−2^	3.008·10^−2^
58	3	2.113	0.905	4.857	3.000	3.391·10^−2^	1.749·10^−2^
59	4	1.153	1.886	2.057	7.000	2.005·10^−2^	3.608·10^−2^
60	3	1.066	0.534	3.200	1.943	2.027·10^−2^	1.142·10^−2^
61	2	1.025	0.575	9.143	3.943	1.967·10^−2^	1.087·10^−2^
62	4	1.655	3.669	4.629	0.686	3.208·10^−2^	1.167·10^−2^
63	3	1.023	1.065	2.829	3.771	1.838·10^−2^	2.091·10^−2^
64	2	0.152	0.856	1.029	3.886	6.669·10^−3^	1.802·10^−2^
65	2	0.842	0.608	3.943	2.514	1.639·10^−2^	1.164·10^−2^
66	3	2.306	1.152	5.400	3.400	3.645·10^−2^	2.125·10^−2^
67	2	1.115	0.321	4.600	0.543	2.115·10^−2^	3.373·10^−3^
68	1	0.603	0.299	4.629	1.771	9.333·10^−3^	6.419·10^−3^
69	3	1.816	0.954	7.200	3.029	3.501·10^−2^	1.662·10^−2^
70	3	0.869	0.553	3.714	2.200	1.815·10^−2^	1.248·10^−2^
71	2	1.176	0.501	7.029	2.429	2.159·10^−2^	9.018·10^−3^
**72**	**4**	**-23.80**	**3.942**	**3.057**	**2.857**	**4.580·10^−2^**	**3.523·10^−2^**
73	3	1.951	0.545	6.686	1.657	3.665·10^−2^	9.593·10^−3^
74	2	0.692	1.231	3.857	0.914	1.336·10^−2^	7.255·10^−3^
75	1	0.571	0.715	2.029	3.114	1.119·10^−2^	1.590·10^−2^
76	2	0.445	0.244	3.057	1.457	8.119·10^−3^	4.863·10^−3^
**77**	**4**	**2.529**	**-11.81**	**6.457**	**1.114**	**4.542·10^−2^**	**1.985·10^−2^**
78	4	5.817	4.253	5.914	2.371	6.424·10^−2^	3.086·10^−2^
79	4	4.019	2.907	4.200	2.143	4.738·10^−2^	2.677·10^−2^
80	4	2.277	1.372	3.514	3.314	3.707·10^−2^	2.742·10^−2^
81	3	0.896	0.560	4.857	2.171	1.857·10^−2^	1.068·10^−2^
82	4	2.150	1.541	4.229	1.629	3.751·10^−2^	2.072·10^−2^
83	2	1.853	0.391	11.29	1.343	3.520·10^−2^	7.224·10^−3^
84	2	1.041	0.499	4.143	1.743	1.976·10^−2^	9.136·10^−3^
**85**	**5**	**2.279**	**1.049**	**8.000**	**3.829**	**4.724·10^−2^**	**2.173·10^−2^**
86	2	1.290	0.296	9.543	1.857	2.513·10^−2^	5.917·10^−3^
87	3	1.241	0.796	3.914	4.000	2.314·10^−2^	1.572·10^−2^
88	3	1.419	1.152	7.171	4.314	2.693·10^−2^	2.308·10^−2^
89	2	0.961	0.422	4.686	1.400	1.837·10^−2^	7.562·10^−3^
90	2	1.356	0.900	6.829	4.314	2.776·10^−2^	1.930·10^−2^

### Evaluation of linearized stiffness

Evaluation of linearized stiffness *k_1i_* in the displacement interval of *u_i_* = 0-5 mm for both directions is depicted in Table 3. The mean linearized stiffness in the present patient group was 3.746±1.163 N·mm^−1^ (min. 1.600 N·mm^−1^, Patient 14; max. 6.543 N·mm^−1^, Patient 61; note that the mean from both directions was considered). The minimum difference between the vertical and horizontal directions in the same patient was 0.086 N·mm^−1^ (Patient 87), and the maximum difference was 9.943 N·mm^−1^ (Patient 83). The mean linearized stiffness in the patient group was 2.562±1.385 N·mm^−1^ for the horizontal and 4.930±1.983 N·mm^−1^ for the vertical direction, respectively. Herewith, the breast stiffness pathology was substantially higher in the vertical than in the horizontal direction.

Hence, neither Boyd's radial stiffness nor the linearized stiffness method is robust enough to fit the entire population as far as the assumptions are concerned. For e.g., in some women, the hemispherical assumption of Boyd is not met and, hence, results show negative stiffness values (see Patients 72 and 77). On the other hand, linearized stiffness does not consider the size of the breast, which could confound the stiffness measurement (a large soft breast would return the same value as a small stiff breast, see Patient 24 with the largest right breast classified by CBE as Class I and Patient 85 with smallest right breast classified by CBE as Class V in Table 3) and it is obvious that it does provide highly different results in the orthogonal directions, which makes the approach unreliable. However, the linearized stiffness method may in the future play a role, for example, in the production of customized individualized underwear and prosthesis. Even now, individualized structures that are wearable can be designed and 3D printed according to the required stiffness.^33^^,^^34^

### Evaluation of elastic modulus

The results of the evaluation of the elastic modulus in *E_i_* individual patients are presented in Table 3. The mean elastic modulus range in the present patient group was 20.79·10^−3^ ± 8.385·10^−3^ N·mm^−2^ (min. 5.895·10^−3^ N·mm^−2^, Patient 20; max. 47.55·10^−3^ N·mm^−2^, Patient 78; mean from both directions was considered). The minimal difference between the vertical and horizontal direction in the same patient was 0.050·10^−3^ N·mm^−2^ (Patient 47), and the maximal difference was 36.32·10^−3^ N·mm^−2^ (Patient 18). The mean elastic modulus in the patient group was 15.21·10^−3^ ± 8.386·10^−3^ N·mm^−1^ in the horizontal direction and 26.36·10^−3^ ± 12.07·10^−3^ N·mm^−1^ in the vertical direction, respectively. *A posteriori*, in many cases, the measurement does not give the same results in both orthogonal directions (*E_x_* ≠ *E_y_*) (Table 3). The assumption of an ideal homogenous and isotropic behavior in the range of *u_max_*, which presumes a close match of measured elastic modulus in both directions (*E_x_* ≅ *E_y_*), was valid only for a fraction of patients (e.g., for Patients 1, 5, 8, 11, 13, 20, etc.). Similar to the previous method, the elastic modulus was substantially higher in the vertical than in the horizontal direction. Generally, the mechanical behavior of the breast is non-homogeneous and anisotropic. To this end, the determination of a single value of elastic modulus using isotropic behavior obtained from two perpendicular directions was found unsuitable for the assessment of breast stiffness pathology.

### Evaluation of the increment of strain energy density

The increment of strain energy density in the present patient group was 2.737±1.110 J·mm^−3^ (min. 1.084 J·mm^−3^, Patient 76, max. 6.155 J·mm^−3^, Patient 85; mean from both directions was considered). The minimum difference between the vertical and horizontal direction in the same patient was 0.012 J·mm^−3^ (Patient 24), and the maximum difference was 3.010 J·mm^−3^ (Patient 22). The mean increment of the strain energy density in the patient group was 2.547±1.140 J·mm^−3^ in the horizontal and 2.927±1.210 J·mm^−3^ in the vertical direction, respectively. The results of the evaluation of the increment of strain energy density Δ*U_i_* for both directions in individual patients are presented in Table 4. The obtained results clearly show that the value of the increment of strain energy density Δ*U_i_* in both directions can be considered consistent $\Delta U\;=\frac{\Delta U_{x}+\Delta U_{y}}{2}\approx \Delta U_{x}\approx \Delta U_{y}$.

**Table 4:** Breast stiffness is classified based on CBE according to the newly designed stiffness scale, evaluation of the increment of strain energy density *U_i,_* and classification of patients based on the energetic approach. All calculated values are calculated in the interval of the displacement of *u_i_* = 0‒5 mm (horizontal direction *i* =*x* and vertical direction *i* = *y)*.

Patient number [-]	Class, based on CBE [-]	∆*U_y_* [J·mm^−3^]	∆*U_x_* [J·mm^−3^]	∆*U* [J·mm^−3^]	Class, based on MMG [-]
1	1	1.429	1.511	1.470	1
2	2	1.346	1.954	1.650	2
3	3	1.793	2.632	2.212	2
4	1	1.670	2.711	2.190	2
5	2	1.677	1.820	1.749	2
6	3	3.179	3.539	3.359	3
7	2	1.873	2.462	2.167	2
8	2	1.669	2.794	2.231	2
9	3	3.273	3.708	3.490	3
10	2	1.688	3.260	2.474	2
11	3	4.209	4.092	4.150	3
12	4	3.741	6.094	4.917	4
13	2	1.353	1.810	1.581	2
14	1	1.154	1.925	1.539	2
15	3	4.595	2.211	3.403	3
16	2	2.459	3.180	2.820	2
17	3	3.075	3.657	3.366	3
18	4	4.137	5.257	4.697	4
19	1	0.763	1.619	1.191	1
20	1	1.163	1.013	1.088	1
21	3	1.447	2.677	2.062	2
22	3	2.151	5.161	3.656	3
23	2	1.401	2.660	2.031	2
**24**	**1**	**1.183**	**1.171**	**1.177**	**1**
25	3	3.131	3.190	3.161	3
26	2	2.028	2.381	2.205	2
27	4	3.460	4.839	4.150	3
28	3	2.375	2.340	2.357	2
29	2	1.877	2.546	2.212	2
30	2	1.766	2.742	2.254	2
31	2	3.752	4.292	4.022	3
32	2	2.541	1.783	2.162	2
33	2	2.504	2.281	2.392	2
34	2	2.869	2.045	2.457	2
35	3	2.352	3.504	2.928	2
36	2	1.288	1.732	1.510	2
37	3	2.674	1.676	2.175	2
38	3	3.347	3.144	3.246	3
39	2	1.459	1.616	1.537	2
40	3	3.196	3.132	3.164	3
41	2	1.367	1.694	1.531	2
42	2	1.665	2.213	1.939	2
43	3	3.045	3.128	3.086	3
44	3	1.888	2.802	2.345	2
45	3	2.940	3.730	3.335	3
46	2	1.611	2.501	2.056	2
47	3	3.063	2.692	2.878	2
48	2	2.299	2.475	2.387	2
49	3	3.022	2.286	2.654	2
50	2	2.544	2.589	2.566	2
51	3	1.562	1.983	1.772	2
52	2	1.544	1.926	1.735	2
53	2	1.703	2.314	2.009	2
54	2	2.206	2.878	2.542	2
55	3	2.616	2.461	2.538	2
56	4	5.003	5.433	5.218	4
57	3	3.046	2.677	2.862	2
58	3	2.232	3.165	2.699	2
59	4	4.578	4.644	4.611	4
60	3	2.855	3.333	3.094	3
61	2	1.087	1.525	1.306	1
62	4	5.277	3.736	4.507	4
63	3	3.099	2.754	2.927	2
64	2	2.186	2.007	2.096	2
65	2	2.306	2.526	2.416	2
66	3	2.668	3.506	3.087	3
67	2	1.455	2.046	1.750	2
68	1	1.340	1.136	1.238	1
69	3	2.132	2.698	2.415	2
70	3	3.801	3.993	3.897	3
71	2	1.297	1.929	1.613	2
72	4	4.333	4.778	4.555	4
73	3	2.384	3.899	3.142	3
74	2	1.629	1.241	1.435	1
75	1	2.766	2.358	2.562	2
76	2	1.032	1.136	1.084	1
77	4	5.274	4.424	4.849	4
78	4	4.545	5.416	4.981	4
79	4	4.627	4.922	4.774	4
80	4	4.788	5.414	5.101	4
81	3	2.072	2.148	2.110	2
82	4	5.133	4.130	4.631	4
83	2	2.143	3.389	2.766	2
84	2	2.008	2.400	2.204	2
**85**	**5**	**5.076**	**7.234**	**6.155**	**5**
86	2	1.259	2.381	1.820	2
87	3	2.481	3.787	3.134	3
88	3	2.761	2.457	2.609	2
89	2	1.750	2.055	1.902	2
90	2	2.648	2.895	2.772	2

## Discussion

Given the unreliable results of the remaining methods (insufficient robustness of Boyd's method due to the hemispherical assumption and differences in orthogonal directions in the evaluation using linearized stiffness and elastic modulus), only the results of the energetic approach are compared with the CBE results. Based on the evaluation results of all four methods, the energetic approach, *id est*, the evaluation of the increment of strain energy density Δ*U*, appears to be the most suitable approach for the automated evaluation of breast stiffness. The obtained results were not affected by possible errors resulting from the idealization of the measured response. Analyzed values of the increment of the strain energy density Δ*U_i_* from both directions (vertical and horizontal) yielded similar results. The consistency of results obtained through the presented energetic approach in both directions led us to analyze the possible correlation of these results with those acquired through CBE.

Therefore, the authors have tried to design the intervals of the increment of strain energy density Δ*U* that would correspond to the stiffness classes proposed in Table 2. The authors postulate the linear classification into five classes. The size of the intervals was approximated to optimize the fit between the increment of strain energy density Δ*U* and classification into stiffness classes based on CBE, using a loop in MATLAB (MathWorks, Natick, MA, USA). The optimization was based on mean Δ*U* values from both directions in each patient; a step of 1.5 J·mm^−3^ was found to provide the best fit, see Tables 4 and 5. The size of these intervals can be changed based on more extensive research with a greater number of cases.

**Table 5:** Intervals of the increment of the strain energy density Δ*U* corresponding to the novel stiffness scale and classification of patients based on CBE and the energetic approach.

Class	Meaning	CBE	*ΔU* [J·mm^−3^]	MMG
I	Easy to examine	8/90 (8.9%)	(0.0, 1.5)	8/90 (8.9%)
II	Well-examinable	37/90 (41.1%)	< 1.5, 3.0)	52/90 (57.8%)
III	Sufficiently examinable	32/90 (35.6%)	< 3.0, 4.5)	18/90 (20.0%)
IV	Difficult to examine	12/90 (13.3%)	< **4.5**, 6.0)	11/90 (12.2%)
V	Non-examinable	1/90 (1.1%)	< 6.0, 7.5)	1/90 (1.1%)

The outcomes of Table 5 indicate that patients in whom the increment of strain energy density was over 4.5 J·mm^−3^ (calculated in the interval of the displacement of *u_i_* = 0‒5 mm and the breast preload *F_0_* = 20 N) are practically unable to perform a valid BSE due to a high breast stiffness. Using this newly created energetic approach method, 13.3% of the cases were selected for more intensive paraclinical examination within the scope of secondary prevention.

**Figure 6:**
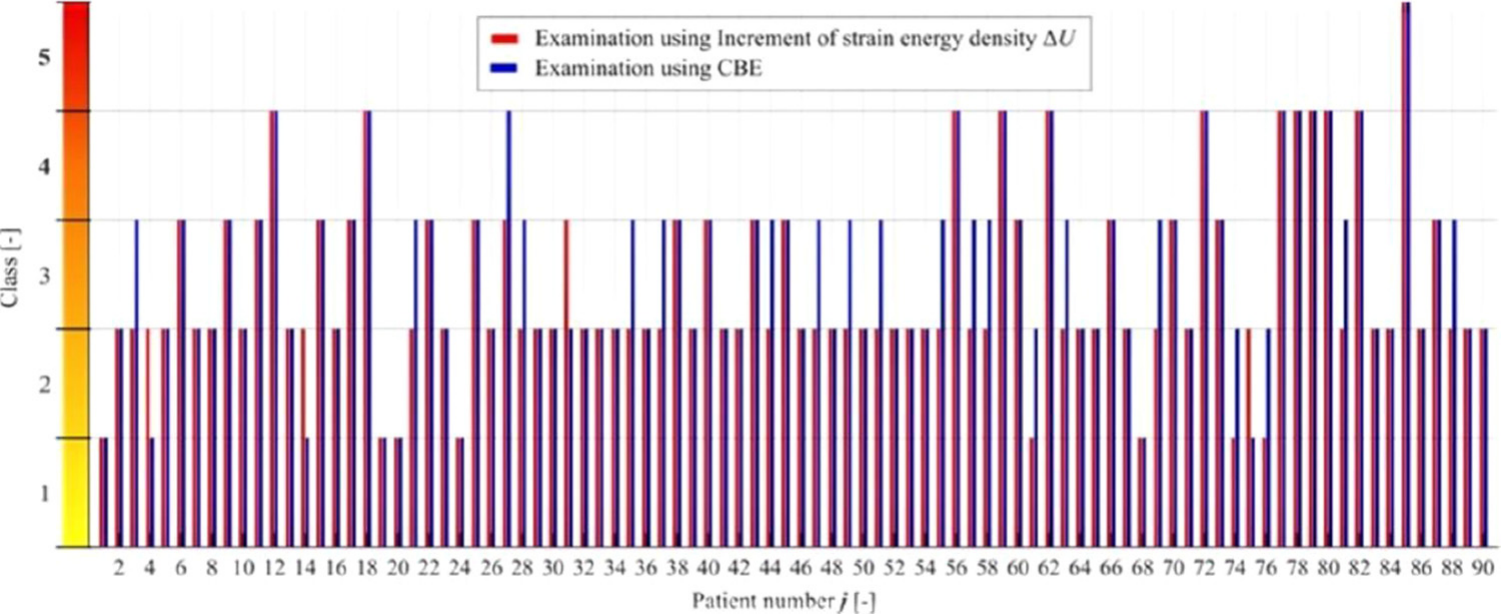
Comparison of the results of the novel stiffness scale classified based on CBE and the energetic approach.

Figure 6 exhibits the goodness of fit between CBE and Δ*U*, with red columns indicating results obtained by computation and blue columns results obtained by CBE in the individual cases.

The result of breast stiffness measurement as measured by MMG was blinded to both examiners until the end of the study. Results by both examiners were in agreement in 93.4% of the cases. Where the subjective measurements differed, the difference was not more than 1 level of subjective stiffness; in such cases, the higher level of stiffness was considered.

As such, breast self-examination plays an essential role in the secondary prevention of breast cancer. Breast cancer etiology is associated with defective tissue structure, which provides particularly good dispositions for malignant tumor growth. To this end, tissue structure changes also lead to a variable X-Ray density, which is already considered to be a risk factor in predictive tools of carcinogenesis. Of note, the rearrangement of the physiological tissue organization very often leads to increased breast stiffness pathology, which also affects carcinogenesis.

The authors postulate that higher breast stiffness also leads to more difficult BSE and relying on this examination method in such patients is, therefore, inappropriate. However, evaluating the feasibility of BSE, be it by the woman herself or by an experienced examiner, is highly subjective. Hence, this study purposed to develop a method for objective assessment of the suitability of BSE depending on breast stiffness and, thus, for finding out if this method is effective in any particular woman; in other words, to determine whether or not the particular female is capable of performing valid BSE. Besides, high stiffness can also mean an alteration of the tissue structure. The authors suggest that individuals with high breast stiffness would benefit from increased frequency of examination by paraclinical methods, which can, to a certain extent, eliminate this handicap.

In the present study, one hundred patients with breast structure unaffected as much as possible were examined (right breasts only). The measurements were performed without the need for X-Ray irradiation on a digital MMG. The results of stiffness, *per se*, obtained by instrumental measurement were compared with those obtained through two independent subjective CBE evaluations by specialists.

Herewith, the authors kindly present four methods for the evaluation of breast stiffness pathology. The first method (Boyd's radial stiffness) is not robust and, in some cases, yields negative stiffness results. The hemispherical simplification of the breast shape does not seem to be a suitable assumption. Although it is necessary to point out that Boyd's research focused rather on the association between breast stiffness and risk of breast carcinoma so the method might have been suitable for their study,^16^ it appears that this method is not universally applicable. The second method (linearized stiffness) approximates the measured response using the least-squares method. This method is the only one of the presented methods disregarding the breast size. The evaluated results differ in the vertical and horizontal directions and, hence, the method is not suitable for comparison with the subjective examination. The third one (the evaluation of the elastic modulus) assumes the isotropic behavior of the material. However, this assumption was confirmed in only a fraction of patients and, therefore, this method was not pursued further, either. The last one (the evaluation of the increment of the deformation energy density) proved to be suitable for comparison with clinical palpation examination, CBE.

There are several advantages to the system presented in this study. First and foremost, the measurement is based on a remarkably simple system. It does not require any X-Ray radiation and can be easily implemented into current mammographic devices. After such modification, current mammographs could include another descriptive variable to be used in preventive and diagnostic algorithms, yielding more complex breast tissue evaluation compared to the traditional mammogram and density variables. After determining stiffness, women with stiff breasts could be referred for more frequent clinical follow-ups, possibly improving secondary breast cancer prevention, and establishing breast stiffness pathology as a risk factor for breast carcinogenesis. The authors suggest that women categorized into stiffness Class IV and V (13.3% of examined women) would be candidates for earlier and/or more frequent paraclinical examination.

It is important to note that not all women may be objectively evaluated by the proposed method. This is especially true for females presenting with some of the exclusion criteria, such as breast illness, hormonal changes, breast malignancy, or breastfeeding. The efficacy of this method in the evaluation of these women should be subject to further research. It is, however, necessary to point out that some generally accepted assumptions about breasts were proven false. The presumption that only young women have stiff breasts is not valid (e.g., Patients 66 or 79, see Tables 1 and 2) and it is not true that breast size affects clinical examination, either (e.g., Patients 24 and 74, see Table 2), although it was noted that breasts with large volumes require more patience during the examination.

An objection can be raised that in this paper, the authors use CBE as a “gold standard” and, therefore, use a subjective method for validation of the proposed objective. This objection is true, but, at the same time, it represents the very reason for this research. The problem is that at present, there is no objective method for breast stiffness evaluation (radiological density and stiffness do not always correlate) and, for this reason, the maximum objectivization the authors could have used as a gold standard was an independent CBE by two experienced physicians. This is, however, non-transferrable to other physicians in other hospitals. Last but not least, introducing an objective method that would be usable everywhere and easily implemented in the current X-Ray instrumentation would, therefore, be a step forward that would allow detailed research on the associations between breast stiffness and breast cancer development, and treatment success, or other clinical issues.

As such, the authors do not claim that the cutoff values for individual categories the authors determined based on the CBEs are final and universally valid; such values will need to be re-established based on a multicentre comparison correlating the CBE results with the results of the objective measurement. As soon as such a method is in place, however, it will be possible to compare objective results between centers and countries.

## Conclusions

The authors used a common digital mammographic device to quantitatively determine breast stiffness on a stiffness scale from Class I to Class V in which Class IV and V negatively influence the validity of BSE. In these women, paraclinical examination methods included in the secondary prevention of breast cancer should be used more frequently. Considering the expected relationship between breast stiffness pathology and breast carcinoma incidence, stiffness measurement may also significantly improve the prediction and early detection of breast malignancy.

By evaluating the measured data, the increment of strain energy density was shown to be the most suitable method of calculation. An excellent match in results of breast stiffness classification according to the designed stiffness scale between MMG and CBE was achieved. In 13.3% of women, breast stiffness Class IV and V were identified. These women should be subject to more intensive (frequent) observation than the rest of the female population as in these women, performing valid BSE is impossible and it has, therefore, no purpose.

Considering small differences between both directions during the evaluation of the increment of strain energy density (an average difference of 30%), using data from only one direction could be sufficient. However, to be able to use this new and simpler approach, for which measurement in one projection would be sufficient, it might be necessary to modify the range of intervals to recalculate subjective stiffness. This correction could accelerate the stiffness evaluation. Implementation of this novel methodology into the MMG examination would yield a method for objective evaluation of breast stiffness, which could be easily done by simply altering the software of MMG machines. Of note, this would provide additional data used to properly identify women with dense breasts who require more frequent examinations in order to improve the early detection of breast carcinoma. Herewith, because of its qualities, this examination method could be implemented into the secondary breast cancer prevention system. *Bene diagnoscitur bene curatur.*

## Data availability

The clinical data used to support the findings of this study are available from the corresponding author upon request.

## Authors' contributions

J.P.: Conceptualization, Data curation, Formal analysis, Investigation, Methodology, Project administration, Resources, Software, Validation, Visualization, and Writing-original draft.

P.M.: Data curation, Formal analysis, Investigation, Methodology, Project administration, Resources, Validation, and Visualization.

I.S.: Investigation, Methodology, Software, Visualization, Supervision, and Writing-review & editing.

A.P.: Conceptualization, Data curation, Formal analysis, Investigation, Methodology, Project administration, Resources, Validation, and Visualization.

J.J.: Investigation, Methodology, Project administration, Resources, Validation, and Visualization.

P.H.: Investigation, Methodology, Project administration, Resources, Validation, and Visualization.

J.R.: Investigation, Methodology, Project administration, Resources, Validation, and Visualization.

D.S.: Investigation, Methodology, Software, Visualization, Supervision, and Writing-review & editing.

J.M.S.J.: Investigation, Validation, Visualization, Supervision, and Writing-review & editing.

## Funding sources

This work was supported by the Faculty of Medicine, University of Ostrava, Ostrava, the Czechia with the grant numbers [SGS02/LF/2016] and [SGS16/LF/2017]; The Ministry of Education, Youth and Sports from the Specific Research Project&VŠB-University of Ostrava, Ostrava, the Czechia with the grant number [SP2021/66]; and the APC was funded by the University of Ostrava, Ostrava, Czechia.

## Conflicts of interest

The authors declare no conflicts of interest.
